# Tuning the supramolecular isomerism of MOF-74 by controlling the synthesis conditions†

**DOI:** 10.1039/c9dt01572h

**Published:** 2019-05-21

**Authors:** Andreea Gheorghe, Inhar Imaz, Jarl Ivar van der Vlugt, Daniel Maspoch, Stefania Tanase

**Affiliations:** Heterogeneous Catalysis and Sustainable Chemistry, Van‘t Hoff Institute for Molecular Sciences, University of Amsterdam Science Park 904 1098 XH Amsterdam The Netherlands s.grecea@uva.nl; Catalan Institute of Nanoscience and Nanotechnology (ICN2), CSIC and Barcelona Institute of Science and Technology Campus UAB Bellaterra 08193, Barcelona Spain; Bioinspired, Homogeneous & Supramolecular Catalysis, Van‘t Hoff Institute for Molecular Sciences, University of Amsterdam Science Park 904 1098 XH Amsterdam The Netherlands; ICREA Pg. Lluís Companys 23 08010 Barcelona Spain

## Abstract

Supramolecular isomerism of metal–organic frameworks (MOFs) is known for several MOF structures, having direct implications on the properties of these materials. Although the synthesis of MOF isomers is mainly serendipitous in nature, achieving controlled formation of a target framework is highly relevant for practical applications. This work discusses the influence of additives and synthesis conditions on the formation of porous isomers containing Zn^2+^ as nodes and 2,5-dihydroxy-1,4-benzenedicarboxylate (dobdc^4–^) as a linker. Using solvent mixtures containing strongly coordinated molecules, *e.g. N*,*N*′-dimethylformamide (DMF) and *N*-methylpyrrolidone (NMP), facilitates the formation of porous structures of type [Zn_2_(dobdc)(S)_*x*_]·*y*S (S = DMF, NMP) which are built from dinuclear Zn_2_(O)_2_(CO_2_)_3_ secondary building units (SBUs) consisting of two different edge-sharing polyhedra with the Zn^2+^ ions in a unsaturated coordinative environment. In the presence of water, the Zn^2+^ dimers are converted to one-dimensional infinite Zn^2+^ chains, in which the number of Zn^2+^-linker bonds increases, therefore giving a hydrolytically more stable coordination environment. The full characterization of the isomers as well as their conversion to the most stable isomer is presented.

## Abstract 2

Controlled synthesis of MOF materials with specific properties is highly relevant for practical applications and it still remains a challenging task. This work highlights the key role played by the solvents and specific modulators in the formation and stability of two isomers of the well-known MOF-74.

## Graphical abstract

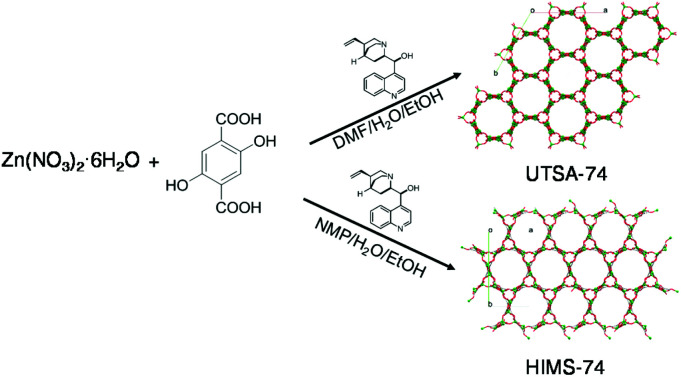

## Introduction

Metal–organic frameworks (MOFs) are porous coordination polymers formed using di- or multitopic organic linkers and metal ions or clusters of metal ions. Currently, more than 20 000 MOF structures are known.^1^ The increased focus on the targeted design and tailor-made synthesis of MOFs emerges from their potential applications in gas storage,^2–4^ molecular separations^3,5,6^ and sensing^7–9^ as well as catalysis.^10–12^ Such broad applications arise from the ability of chemists to fine-tune these materials, even at the atomic level.^13^ Tailoring MOF properties for specific applications can be achieved by making specific topologies^11^ and/or introducing key functional groups within the framework.^14^ Important parameters in controlling the network connectivity are not only the nature of the metal ions and organic linkers used but also the synthesis parameters, *e.g.* temperature, pressure, reaction time and type of reaction vessel.^13,15^ At the same time, the compositional parameters, *i.e.* molar ratio, solvents or solvent mixtures, the addition of modulators, the pH and the reactants’ concentrations also play key roles.^13,15^

The main goal in MOF synthesis is the formation of molecular frameworks in a controlled manner, which is often challenging because of the many synthetic parameters involved. An enticing approach to directing the structure is to use modulators. The most common types of modulators are coordination modulators that are added in excess directly to the reaction mixture. They are usually monocarboxylic acids (*e.g.* formic,^16–18^ acetic,^16,18–20^ and benzoic acids^17,21,22^ as well as aminoacids^23,24^). They act as competitive coordination agents, regulating the pore size and the morphology of the crystals. Modulators play a key role in the synthesis of Zr-MOFs, facilitating the formation of Zr_6_O_4_(OH)_4_ clusters and therefore the growth of the crystals.^22,25^ They also slow down the crystal growth rate, therefore avoiding the fast precipitation of amorphous phases.^20,26,27^ Kaskel *et al.*^28^ reported that the amount of acetic acid used as modulator in the synthesis of C_72_H_36_O_32_Zr_6_ (DUT-52) has a structure-directing effect because it reduces the connectivity of the [Zr_6_O_4_(OH)_4_]^12+^ entity from 12 to 8 or 6, leading to frameworks with different structural topologies.^28^ Modulation can also induce the formation of defects that are responsible for enhanced gas uptake,^29,30^ proton conduction^31^ or catalytic activity.^32–34^ Shafir *et al.*^24^ showed that l-proline is an efficient modulator in the synthesis of Zr MOFs with increased particle size.

The isostructural MOFs known as M-MOF-74^35^ are extensively studied due to the presence of unsaturated coordination sites at metal centers,^35,36^ their versatility of being prepared with different divalent ions^35^ and their high stability under ambient conditions and in the presence of water.^37,38^ Among them, Zn-MOF-74 is a very effective material for gas storage^36^ and molecular separations^39^ and it is also an active catalyst in several organic transformations.^40,41^

**Scheme 1:**
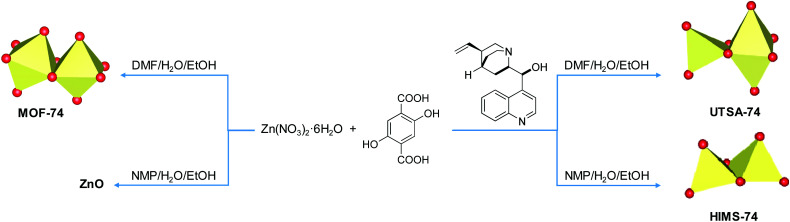
Influence of the synthetic conditions on the formation of secondary building units of porous molecular networks formed from Zn^2+^ as the nodes and 2,5-dihydroxy-1,4-benzenedicarboxylate (dobdc^4–^) as a linker.

Using modulators is not a common approach in the synthesis of the MOF-74 series. Only Li *et al.*^42^ reported that the size and inherent defects of the nanocrystals of Co-MOF-74 can be tuned by employing salicylic acid as the modulator. Our aim was to study the role of *Cinchona* alkaloid derivatives, (+)-cinchonine (CN) and (−)-cinchonidine (CD) as coordination modulators in the synthesis of Zn-MOF-74, with the final goal to design chiral frameworks. We hypothesised that the chiral molecules CN and CD would coordinate to the Zn^2+^ ions during the crystallisation of Zn-MOF-74, thereby directing the handedness of the molecular framework. Bu *et al.*^43^ used *Cinchona* alkaloids to prepare a homochiral In^3+^ dicarboxylate framework, (Me_2_NH_2_)[In(thb)_2_]·*x*DMF (known as ATF-1P, H_2_thb = thiophene-2,5-dicarboxylic acid), using CD as the chiral additive.^44^ The chiral alkaloid binds to the In^3+^ ion and directs the handedness of the framework.^44,45^ The framework does not belong to a chiral space group due to the racemic distribution of the helical chains. Based on the structural similarities between the chiral ATF-1P^43^ framework with large 1D channels of *ca*. 13 Å,^43^ which are formed upon the interaction with chiral *Cinchona* alkaloid modulators, and the MOF-74 framework, which consists of 1D channels of *ca.* 11 Å, we hypothesised that chirality transfer might be achieved upon the coordination of CN or CD *via* amine and hydroxyl groups to the Zn^2+^ ions of MOF-74. Furthermore, the alkaloids can also interact with the framework walls *via* hydrogen bonding interactions. Indeed, Prochowicz *et al.*^46^ reported that *Cinchona* alkaloids lead to chiral coordination polymers using noncovalent interaction driven self-assembly processes. However, we found that using *Cinchona* alkaloids facilitates the formation of a new Zn-MOF-74 isomer, namely, [Zn_2_(dobdc)(NMP)]·⅓NMP (termed HIMS-74 in this work), where dobdc is 2,5-dioxido-1,4-benzenedicarboxylate and NMP is *N*-methylpyrrolidone (Scheme 1). Here, we report the controlled assembly and crystallisation of HIMS-74 and compare its structural features with the previously reported isomers, *e.g.* MOF-74 ^5^ and UTSA-74.^5^ The role of the solvent in the stabilisation of specific framework is also discussed.

## Results and discussion

### Influence of the reaction conditions on the synthesis of MOF-74 isomers

**Fig. 1:**
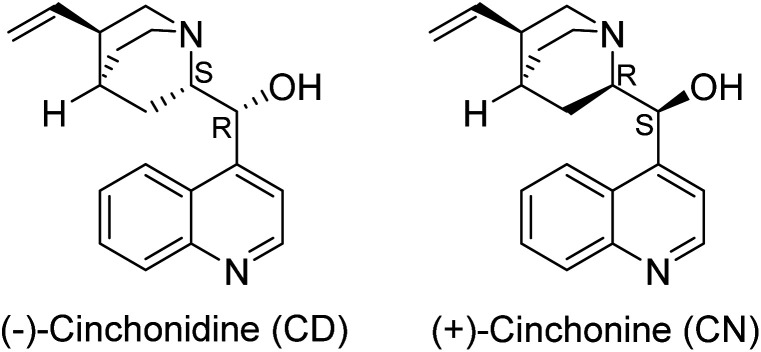
The structure of the *Cinchona* alkaloid modulators, indicating the different configurations of the chiral substituted carbon atom centres.

The Zn^2+^ ion in MOF-74 has one coordinatively unsaturated site, which is occupied by an exchangeable solvent molecule. This site is also available for the coordination of *Cinchona* alkaloid molecules. Therefore, we studied the role of both alkaloids and solvent in the hydrothermal reaction of Zn^2+^ with 2,5-dioxido-1,4-benzenedicarboxylic acid (H_4_dobdc). Fig. 1 shows the alkaloids used in this study.

**Fig. 2:**
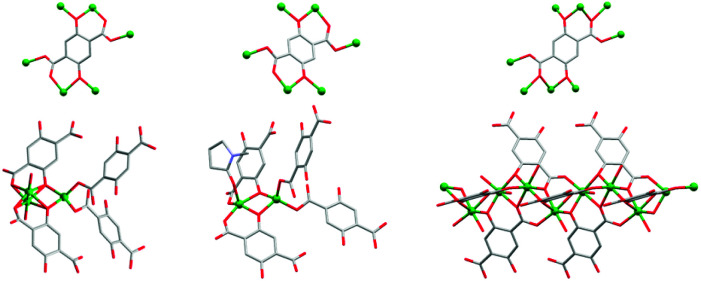
The coordination of dobdc^4–^ to the Zn^2+^ ions and the coordination geometries of the Zn^2+^ ions in UTSA-74 (left), HIMS-74 (middle) and MOF-74 ^47^ (right). The hydrogen atoms and the guest solvent molecules were omitted for clarity. Only the oxygen atom is represented for the disordered coordinated solvent molecules.

Reacting Zn(NO_3_)_2_·6H_2_O, H_4_dobdc (molar ratio Zn^2+^ : H_4_dobdc of 3 : 1) and CN or CD (1 or 3 equiv.) under hydrothermal conditions in two different solvent mixtures (DMF/EtOH/H_2_O or NMP/EtOH/H_2_O *v*/*v*/*v* ratio of 20/1/1) afforded six solid crystalline materials. Single-crystal XRD analysis performed on crystals isolated from DMF/EtOH/H_2_O reaction mixtures revealed the formation of the compound [Zn_2_(dobdc)(solvent)_2_]·*x*H_2_O·*y*DMF (solvent = H_2_O or DMF), which crystallises in the rhombohedral *R*3̄*c* space group and has a 3D porous structure. The asymmetric unit contains two crystallographically independent Zn^2+^ centres and one dobdc^4–^ linker (Fig. 2, left). One Zn^2+^ coordination sphere is composed of four donor atoms from four fully deprotonated linkers, *i.e.* two phenolate oxygens and two carboxylate oxygens, forming a distorted tetrahedral geometry. The Zn–O bond lengths vary from 1.926(5) to 1.957(5) Å. Zn has an octahedral geometry, being surrounded by two phenolate oxygens, two carboxylate oxygens and two oxygen atoms from the solvent molecules (due to the high disorder of the molecular structure, it cannot be determined if they belong to water or DMF molecules). In this Zn^2+^, the Zn–O bond lengths vary from 1.959(5) to 2.120(6) Å. The organic linker binds one Zn^2+^*via* carboxylate oxygen, while it binds another Zn^2+^ as a chelate *via* carboxylate and phenolate oxygen, forming six-membered rings. All the crystallographic data indicate that this structure is identical to the UTSA-74 structure reported earlier.^5^

**Fig. 3:**
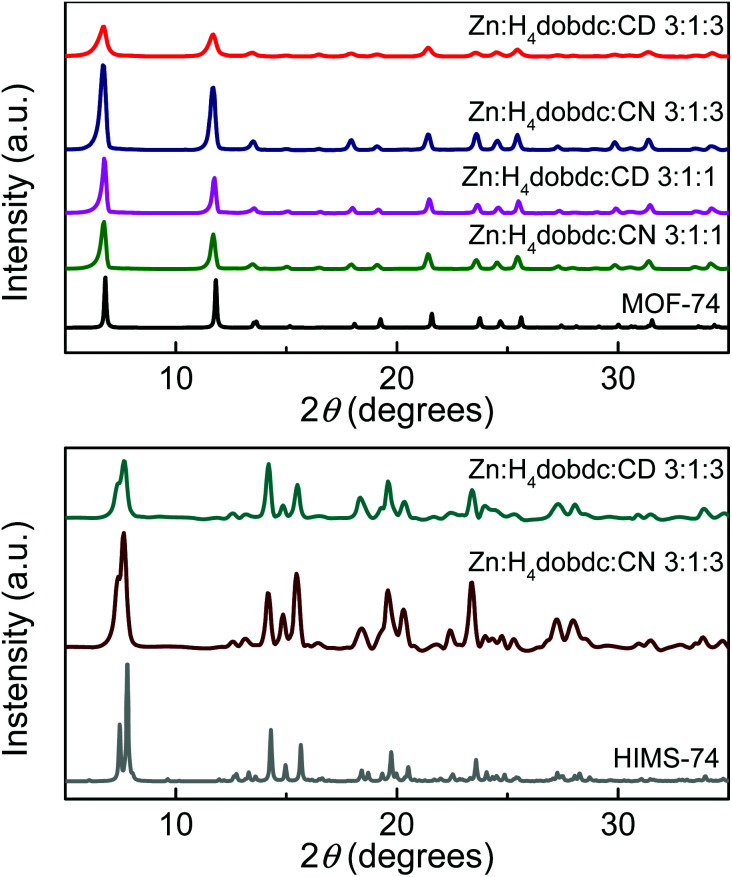
PXRD patterns of the dried materials obtained in DMF (top) and NMP (bottom), and the simulated PXRD patterns for MOF-74 ^47^ and HIMS-74.

Using a polarising optical microscope, the analysis of the materials obtained in DMF/EtOH/H_2_O solvent mixtures revealed UTSA-74 crystals as well as chiral alkaloid modulators as aggregates in solution. Therefore, the isolated solid materials were washed thoroughly with the synthesis solvent mixture to fully remove the alkaloids and then all materials were dried under ambient conditions. Notably, the PXRD patterns of these samples matched perfectly the simulated pattern of MOF-74 topology (Fig. 3). As indicated by PXRD, the conversion of UTSA-74 to MOF-74 is quantitative and it is most likely caused by the atmospheric humidity due to the prolonged air exposure during the drying process. Indeed, the water-induced transformation of UTSA-74 to MOF-74 was also reported by Ameloot *et al.*^48^

The formation of UTSA-74 in our work is likely facilitated by the modulating effect of the alkaloids or the DMF/H_2_O/EtOH solvent mixture used or perhaps a combination of both. To further test the role of the alkaloid during the crystallisation process of UTSA-74, we performed the synthesis in the absence of the alkaloid. In this case, however, no solid material was obtained. This result clearly demonstrates that the CN and CD alkaloids play a key role in the formation of the UTSA structure. Furthermore, using XYZ spatial descriptors, we calculated the length of the alkaloids as 11.8 × 9.0 × 7.2 Å^3^ for CD and 11.8 × 9.3 × 7.3 Å^3^ for CN. This indicates diffusion restrictions for the alkaloids within the 8.81 Å pores of UTSA-74 ^49^ (Fig. S1†), thereby explaining the absence of chirality in the isolated molecular structures.

Our results show that the unstable UTSA-74 phase forms prior to that of MOF-74 in the presence of *Cinchona* alkaloid modulators. Therefore, subsequent studies aimed at understanding the role of the solvent mixture in stabilising the structure. We hypothesised that the solvent mixture may also act as a modulator in our synthetic procedure, a phenomenon also frequently reported.^15,50,51^ Consequently, we replaced DMF by the structurally very different *N*-methylpyrrolidone (NMP), which is known to lead to internal shearing stress and formation of a chiral framework, in the case of MOF-5.^52^ We envisioned that the dobdc^4–^ linker could exhibit a twisting behaviour upon interaction with the NMP molecules as observed for the benzene-1,4-dicarboxylate linker (bdc^2−^) in MOF-5. However, replacing DMF with NMP and using a molar ratio for Zn^2+^ : dobdc^4–^ : alkaloid of 3 : 1 : 3 led to a new structure, namely, [Zn_2_(dobdc)(NMP)]·⅓NMP (hereafter denoted as HIMS-74).

HIMS-74 crystallises in the monoclinic *P*2_1_/*a* space group. Its asymmetric unit contains two crystallographically independent Zn^2+^ ions. One Zn^2+^ ion is tetracoordinated (Fig. 2, middle), being surrounded by two phenolate oxygens and two carboxylate oxygens, forming a distorted tetrahedral geometry. The Zn–O bond lengths vary between 1.926(3) and 1.970(3) Å. The second Zn^2+^ centre is pentacoordinated, with the coordination sphere defined by two carboxylate oxygens, two phenolate oxygens and the oxygen of the coordinated NMP solvent molecule. The NMP is oriented towards the channels of the framework and its position imposes a distorted square pyramidal geometry. In this case, the Zn–O bond length varies from 1.926(3) to 2.092(3) Å. The dobdc^4–^ linkers are hexadentate coordinated, bridging the two independent Zn^2+^ ions *via* the phenoxo group (Fig. 2, middle), whilst the carboxylate groups are bridging Zn^2+^ ions of the same type.

**Fig. 4:**
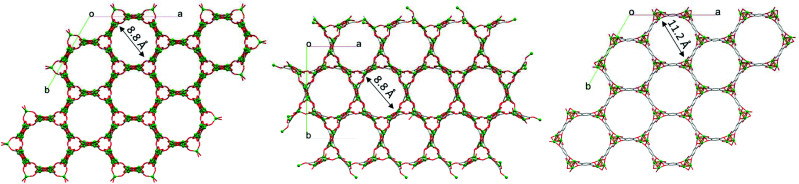
View of the 3D structures of UTSA-74 (left), HIMS-74 (middle) and MOF-74 (right) along the *c* axis. Hydrogen atoms, and guest and coordinated solvent molecules are omitted for clarity.

The dinuclear Zn_2_(O)_2_(CO_2_)_3_ secondary building units (SBUs) consist of two edge-sharing polyhedra which are linked to each other *via* the carboxylate oxygen donors of the dobdc^4–^ linkers to form a 3D network with 1D channels (Fig. 4). Notably, the molecular structure of HIMS-74 is built up from dinuclear SBUs as also observed in the case of UTSA-74 and therefore their 3D molecular structures are very similar (Fig. 4). The main difference is that the SBUs of HIMS-74 consist of a tetrahedron and a square pyramid whilst the UTSA-74 SBUs are composed of a tetrahedron and an octahedron. This reflects the critical role of NMP in stabilising the square pyramidal geometry for the Zn^2+^ ion as compared to DMF, which facilitates the stabilisation of the octahedral geometry.

The PXRD patterns (Fig. 3, bottom) of the crystalline solids isolated from NMP/H_2_O/EtOH solvent mixture show an excellent matching with the calculated pattern using the single-crystal XRD data of HIMS-74. To understand the role of the alkaloids in the synthesis of HIMS-74, we also performed the synthesis in their absence. Interestingly, the solid materials obtained in these reactions proved to be ZnO nanoparticles, as indicated by PXRD (Fig. S2†). Moreover, performing the synthesis using one equivalent of alkaloids does not facilitate the formation of the HIMS-74 structure. Therefore, we believe that both the alkaloids and the solvent mixture play key roles in the crystallization process of HIMS-74.

**Fig. 5:**
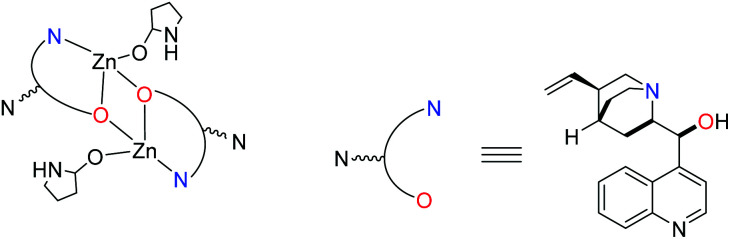
Possible coordination of the *Cinchona* alkaloid to the Zn^2+^ ions which precedes the formation of HIMS-74 SBUs.

**Fig. 6:**
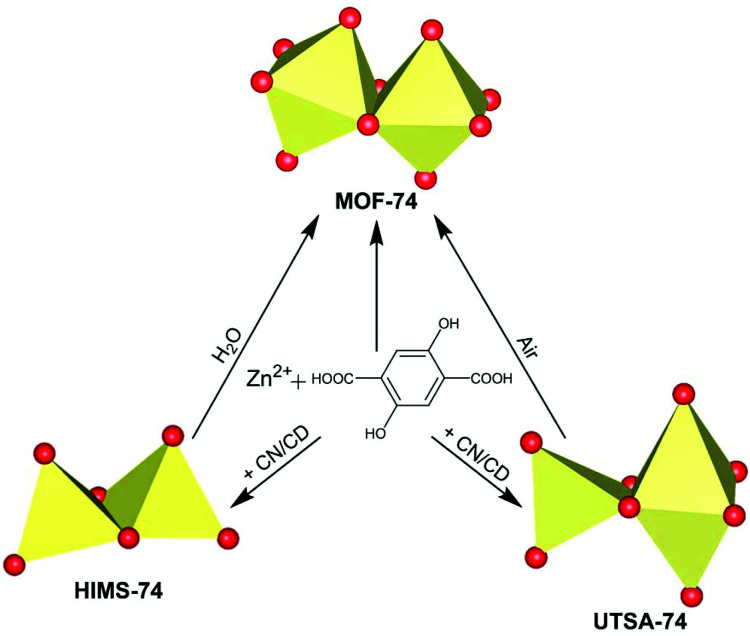
Role of the synthetic conditions in the formation of MOF-74 isomers.

Our studies indicate that some weak interactions between the alkaloids and the SBUs of UTSA-74 and HIMS-74 may favour the formation of dinuclear SBUs as compared to the helical rods present in MOF-74. However, further investigations are necessary to determine the nature of these types of interactions. No induction of chirality using *Cinchona* alkaloids as modulators was observed. This suggests that the interaction of the alkaloid with the porous framework is weak at best. Under hydrothermal conditions, the alkaloid (p*K*_a_1__ = 4.1 and p*K*_a_2__ = 8.2 for CN and p*K*_a_1__ = 4.0 and p*K*_a_2__ = 8.2 for CD)^53^ is easily deprotonated and therefore can coordinate *via* its N and O donor atoms (amine and hydroxyl groups) to the Zn^2+^ ions.^46^ Thus, it is likely that the precursors of the HIMS-74 SBUs are doubly bridged chiral dinuclear units (see Fig. 5 for details) that prevent the formation of helical rods as observed for MOF-74. However, the increase in the basicity in the reaction mixture due to NMP decomposition causes the full deprotonation of the H_4_dobdc linker (p*K*_a_1__ = 12.20 and p*K*_a_2__ = 26.67).^54^ The hexadentate dobdc^4–^ replaces the *Cinchona* alkaloid and forms HIMS-74 achiral SBUs (Fig. 6).

### Phase transformations

As discussed above, the UTSA-74 isomer is not stable and converts to MOF-74 under ambient conditions or in the presence of water. In UTSA-74, there are two types of coordination polyhedra for Zn^2+^ ions, tetrahedron and octahedron, whilst both Zn^2+^ ions have an octahedral geometry in MOF-74. The SBUs of UTSA-74 consist of Zn_2_(O)_2_(CO_2_)_4_ units connected by chains of dobdc^4–^ linkers.^48^ MOF-74 contains helical chains of edge-sharing ZnO_6_ octahedra that are interconnected *via* dobdc^4–^ linkers.^47^ Ameloot *et al.*^48^ attributed the formation of the discrete SBUs in UTSA-74 to a stabilising effect of the DMSO solvent. However, Chen *et al.*^5^ synthesised UTSA-74 in DMF/H_2_O, a similar solvent mixture used for preparing MOF-74.^47^ Our studies show that both the solvent mixture (DMF/H_2_O/EtOH) and the *Cinchona* alkaloids control the formation of UTSA-74. All these results indicate that the formation of the UTSA-74 framework is very complex and depends on many more factors, including the anions present in the reaction mixture (*e.g.* CH_3_COO^−^)^48^ as well as the temperature and reaction time (*e.g.* 72 hours).^48^

As proposed by Ameloot *et al.*,^48^ it is important to consider the potential formation of UTSA-74 in the nucleation and growth process of MOF-74. This implies to consider a sequential stepwise model, known as Ostwald's Rule of Stages,^55^ rather than a classical nucleation and growth model. A careful selection of the reaction parameters and an in-depth morphological analysis of the single crystals formed are required to understand such a complex phase transition. The Zn-UTSA-74 single crystals can be easily distinguished from those of Zn-MOF-74 by analysing their morphology using polarised light microscopy. Initial studies reported that the Zn-UTSA-74 crystallises as not only yellow rod crystals^5^ but also hexagonal^48^ crystals were observed. *In situ* PXRD and SEM analysis of the Zn-UTSA-74 to Zn-MOF-74 phase transitions have shown that the nucleation and growth of the Zn-MOF-74 occurs at the surface of hexagonally shaped UTSA-74 crystals.^48^ This indicates that the MOF-74 phase is thermodynamically more stable. The conversion of the Zn^2+^ dimers of UTSA-74 to the one-dimensional infinite Zn^2+^ chains in MOF-74 increases the number of Zn^2+^-linker bonds from four to five, giving a hydrolytically more stable coordination environment.^48^

HIMS-74 is a highly stable structure under ambient conditions and it does not undergo any structural changes. Attempts to replace the strongly coordinated NMP molecules have revealed that exposing HIMS-74 to polar organic solvents (*e.g.* CH_3_OH) leads to a loss in crystallinity and partial or complete transition to UTSA-74 is likely to occur (Fig. S3†). SEM analysis taken after immersion of HIMS-74 in CH_3_OH (6 days) indicates that the morphology is no longer homogeneous and a wide distribution of crystal sizes is obtained (Fig. S4†). TGA-DSC analysis on the recovered sample also showed that no NMP solvent was present (Fig. S5†); however, N_2_ adsorption studies indicated the lack of porosity in this material (Fig. S6†). The integrity of the HIMS-74 framework is retained in acetone (Fig. S3†); however, NMP is not replaced in the sample as shown in the TGA-DSC analysis (Fig. S5†). To further test the stability of HIMS-74 in water, the solid crystalline material was immersed in water for 20 h. PXRD and SEM studies indicate that HIMS-74 is converted to MOF-74 (Fig. S7 and S8†). These results show that HIMS-74 also undergoes a water-mediated isomerization to MOF-74 and possibly UTSA-74, but the HIMS-74 to MOF-74 phase transition is faster than that of HIMS-74 to UTSA-74 (Fig. S7†).

Unlike UTSA-74, HIMS-74 is stable upon separation from the mother liquor and drying in air, indicating that NMP has a stabilizing effect due to its strong coordination to the Zn^2+^ ions. Moreover, the close orientation of the coordinated NMP molecules towards the aromatic linkers in the single-crystal XRD analysis indicates a bonding *via* CH⋯π interactions (*ca.* 3.2 Å). One non-coordinated NMP molecule is also in close contact with the coordinated NMP and it interacts *via* moderate hydrogen bonding through the carbonyl O atoms, with a CH⋯O bond distance of 2.6–2.8 Å. Earlier studies^52,56^ also showed that the molecular size and the chemical nature of NMP give rise to structural transformation, *e.g.* chiral transformation in MOF-5.

MOF-74 is intensely studied for gas storage,^57,58^ molecular separations^59^ and catalysis applications.^60,61^ Notably, MOF-74 also shows a very high CO_2_ uptake due to the presence of three different CO_2_ adsorption sites.^62^ The first one is based on the electrostatic interaction between the open metal site and CO_2_, the second one is related to the van der Waals interaction of the CO_2_ with the host framework, and the third one is only related to the filling of the MOF channels. Although UTSA-74 has the same number of open metal sites per SBU as MOF-74, a smaller CO_2_ uptake was observed for UTSA-74 as compared to MOF-74.^5^ This was ascribed to the loose packing of the CO_2_ molecules in UTSA-74 (7.5 Å O⋯O) as compared to MOF-74 (3.6 Å O⋯O)^5^ and also to the slightly smaller pore size. The available open sites in HIMS-74 indicate two adsorption sites per SBU unit, similar to UTSA-74, and therefore similar CO_2_ uptake is expected. Nevertheless, we have not been able to verify it experimentally because the adsorption studies indicated that HIMS-74 is not porous (Fig. S9 and S10†). This is due to the unsuccessful removal of the coordinated NMP molecules without loss of sample crystallinity (Fig S11†) and it clearly demonstrates the key role of NMP in stabilizing the HIMS-74 framework.

## Conclusions

This work demonstrates that the designed synthesis of MOFs remains a complex process and that the starting materials, synthesis solvents and the modulators play critical role in the stabilisation of various MOF structures. Specifically, we showed that using *Cinchona* alkaloids as modulators as well as the choice of solvent have direct implications for the crystallisation of different MOF-74 isomers. In addition to the previously known MOF-74 and UTSA-74 frameworks, a new HIMS-74 structure is formed when using NMP instead of DMF as coordinating solvent molecules. Unlike UTSA-74, the HIMS-74 isomer is more stable under ambient conditions and it undergoes a solvent-mediated isomerisation to MOF-74 only by immersing it in water. A comparative study of the adsorption properties of the three isomers was not possible because HIMS-74 is not stable upon solvent removal. This work highlights a new strategy to control framework formation when using Zn^2+^ nodes and dobdc^4–^ linkers. It can also be applied to other MOF systems, thus affording the facile synthesis of structural isomers.

## Experimental

### Materials and methods

All chemicals were purchased from commercial suppliers and used without further purification. The bulk synthesis of UTSA-74 used for the phase transition studies was performed following a reported procedure.^48^ The MOF-74 sample for SEM studies was prepared using a procedure from the literature.^63^

#### General procedure for synthesis of compound [Zn_2_(dobdc)(DMF)_2_]·*x*H_2_O·*y*DMF (UTSA-74)

A mixture of Zn(NO_3_)_2_·6H_2_O (0.089 g, 0.3 mmol), 2,5-dihydroxyterephthalic acid (0.019 g, 0.1 mmol), and (−)-cinchonidine alkaloid (0.088 g, 0.3 mmol) and a solvent mixture of DMF : H_2_O : EtOH (20 : 1 : 1 v : v : v, 15 mL) were placed in a 20 mL Teflon screw-capped Duran™ Pyrex tube. The mixture was sonicated and then placed in a preheated oven and kept for 48 h at 120 °C. The reaction mixture was then cooled down to room temperature and the solid crystalline material was filtered and washed three times with the synthesis solvent. IR (KBr, cm^−1^): 3434 (m, b), 2959 (w), 2927 (w), 1656 (s, s), 1559 (s, s), 1511 (w), 1450 (m, s), 1410 (s, s), 1363 (w), 1307 (w), 1239 (m, s), 1196 (m, s), 1114 (w, s), 1063 (w), 910 (w), 879 (m, s), 815 (m, s), 676 (w), 635 (w), 578 (w, s).

#### General procedure for synthesis of compound [Zn_2_(dobdc)(NMP)]·⅓NMP (HIMS-74)

A mixture of Zn(NO_3_)_2_·6H_2_O (0.089 g, 0.3 mmol), 2,5-dihydroxyterephthalic acid (0.019 g, 0.1 mmol), and (−)-cinchonidine alkaloid (0.088 g, 0.3 mmol) and a solvent mixture of NMP : H_2_O : EtOH (20 : 1 : 1 v : v : v, 15 mL) were placed in a 20 mL Teflon screw-capped Duran™ Pyrex tube. The mixture was sonicated and then placed in a preheated oven and kept for 48 h at 120 °C. The reaction mixture was then cooled down to room temperature and the solid crystalline material was filtered and washed three times with NMP and one time with acetone. IR (KBr, cm^−1^): 3436 (m, b), 2958 (w), 2927 (w), 2873 (w), 1662 (m, s), 1646 (m, s), 1535 (s, s), 1469 (m, s), 1434 (m, s), 1410 (m, s), 1383 (m, b), 1304 (w, b), 1256 (m, s), 1234 (m, s), 1214 (m, s), 1119 (w, b), 1031 (w), 990 (w), 891 (m, s), 825 (m, s), 802 (m, s), 670 (w), 610 (m, s). C, H, N analysis (%): calcd for [Zn_2_(dobdc)(NMP)]·⅓NMP (Zn_2_C_15.33_H_15.33_N_1.33_O_7.33_) C 38.54, H 3.09, N 4.08; found C 38.72, H 3.54, N 4.14.

### Physical characterisation

Powder X-ray diffraction (PXRD) patterns were recorded on a Rigaku MiniFlex II X-Ray diffractometer using Cu Kα radiation (*λ* = 1.5406 Å). The X-ray tube was operated at 30 kV and 15 mA. Measurements were carried out in an angle (2*θ*) range of 3–60° with a turning speed of 2° min^−1^. PDF card no. 2300112 was used as a reference for the PRXD of ZnO. Thermogravimetric analysis (TGA) and differential scanning calorimetry (DSC) were performed in the 35–800 °C range at 5 K min^−1^ using a NETZSCH Jupiter® STA 449F3 instrument. The measurements were carried out under a flow of argon (20 mL min^−1^) and protective argon (20 mL min^−1^). N_2_ and CO_2_ adsorption isotherms were measured on a Thermo Scientific Surfer instrument at 77 K and 273 K, respectively. The as-synthesised HIMS-74 was pre-treated in a vacuum with 1° min^−1^ heating from room temperature to 150 °C and a hold of 2 h. The methanol exchanged HIMS-74 sample was heated under vacuum from room temperature to 80 °C with a ramp of 2° min^−1^ and a hold of 1 h, then heating to 120 °C with 4° min^−1^ and 1 h hold, and lastly to 220 °C with 2° min^−1^ and a final hold of 12 h. Scanning electron micrographs were obtained using a field emission scanning electron microscope FEI-Verios 460. The samples were previously sputtered with a 20 mm thick Au layer using a Leica EM ACE600 Double sputter coater.

Single-crystal X-ray diffraction (SCXRD) data of HIMS-74 and UTSA-74 analogue were collected at 100(2) K in the BL13-XALOC beamline^64^ at the ALBA synchrotron, on a single-axis goniometer with a Pilatus 6 M detector using a monochromatic X-ray beam (*λ* = 0.72656 Å). The data frames were integrated and scaled using the XDS software package.^65^ Absorption correction was not applied. The structure was solved by direct methods and subsequently refined by correction of *F*^2^ against all reflections, using SHELXT2013 and SHELXL2013 within the WinGX package.^66–68^ Both structures contain some disordered molecules. Attempts to adequately model the disordered molecules were unsatisfactory; therefore, the PLATON/SQUEEZE routine was applied to mask out the disordered electron density.^69^

## Conflicts of interest

There are no conflicts to declare.

## Supplementary Material

DT-048-C9DT01572H-s001

DT-048-C9DT01572H-s002
