# Supplementary material for: Tuning the supramolecular isomerism of MOF-74 by controlling the synthesis conditions
Source: Dalton Trans. 2019 May 21;48(27):10043–50. doi: 10.1039/c9dt01572h (PMC8612727; doi:10.1039/c9dt01572h)
Supplement: DT-048-C9DT01572H-s001 [file DT-048-C9DT01572H-s001.pdf]

Electronic Supplementary Material (ESI) for Dalton Transactions.
This journal is © The Royal Society of Chemistry 2019

Supplementary information

Tuning the supramolecular isomerism of MOF-74 by controlling the synthesis conditions

Andreea Gheorghe [a], Inhar Imaz [b], Jarl Ivar van der Vlugt [c], Daniel Maspoch [b, d], and Stefania Tanase*[a]

[a.] Heterogeneous Catalysis and Sustainable Chemistry, van 't Hoff Institute for Molecular Sciences, University of Amsterdam, Science Park 904, 1098 XH, Amsterdam, The Netherlands.

[b.] Catalan Institute of Nanoscience and Nanotechnology (ICN2), CSIC and Barcelona Institute of Science and Technology, Campus UAB, Bellaterra 08193, Barcelona, Spain.

[c.] Bioinspired, Homogeneous & Supramolecular Catalysis, van 't Hoff Institute for Molecular Sciences, University of Amsterdam, Science Park 904, 1098 XH Amsterdam, The Netherlands.

[d.] ICREA, Pg. Lluís Companys 23, 08010 Barcelona, Spain.

*To whom correspondence should be addressed.

E-mail: s.grecea@uva.nl

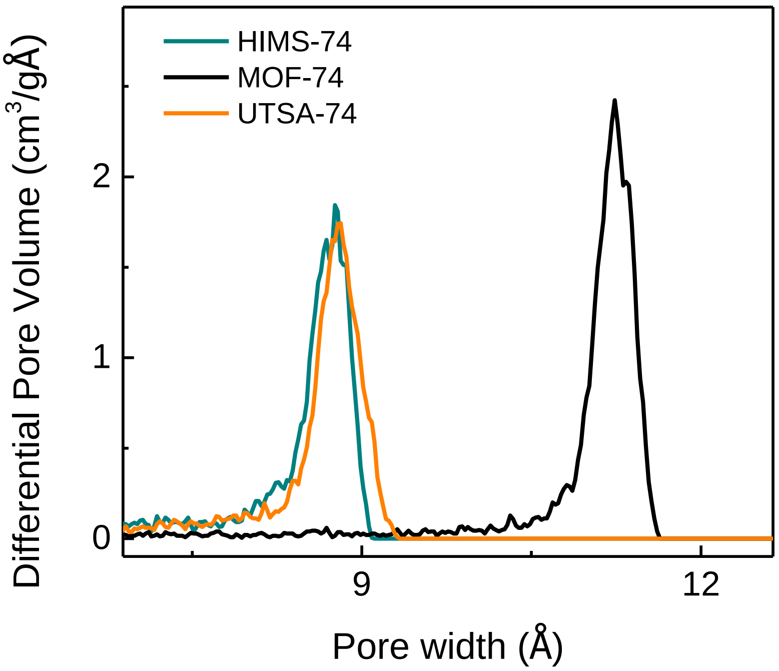

Fig. S1 Pore size distributions (PSDs) calculated from the single-crystal crystallographic data using iRASPA[1] for UTSA-74, HIMS-74 and MOF-74[2].

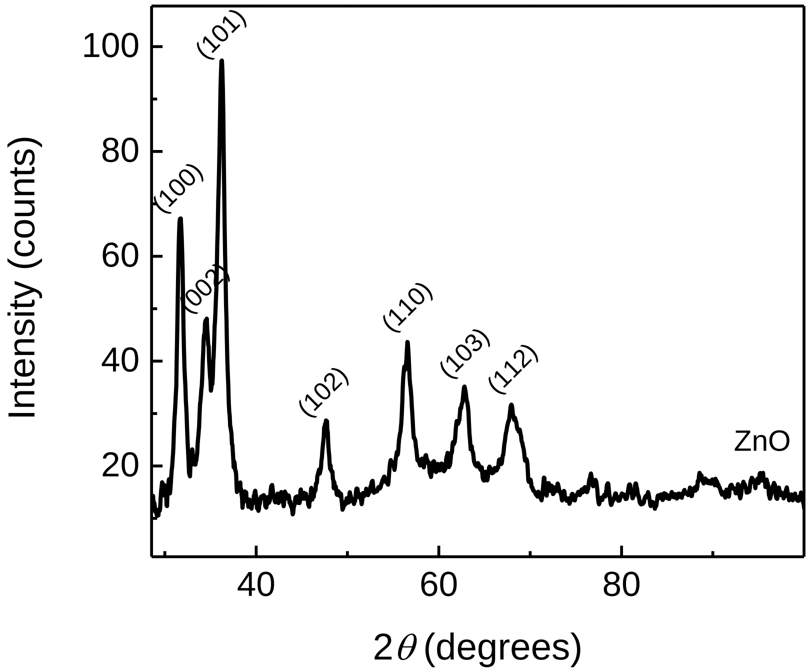

Fig. S2 PXRD pattern of the material obtained by reacting $Zn(NO_3)_2 \cdot 6H_2O$, H_4dobdc, molar ratio Zn:linker 3:1, in NMP/EtOH/H_2O (*v/v/v* 20/1/1), under solvothermal conditions, in the absence of *cinchona* alkaloid.

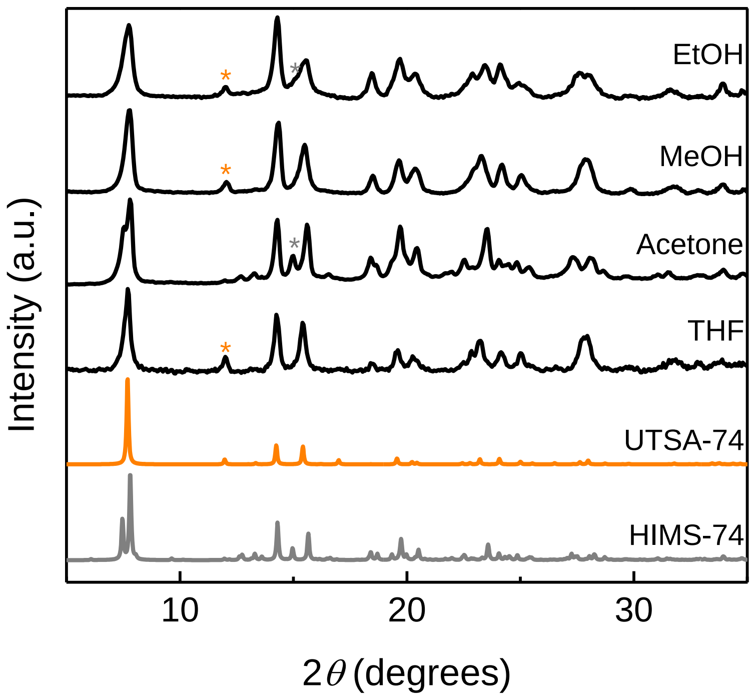

Fig. S3 PXRD pattern of the simulated UTSA-74 and HIMS-74 and HIMS-74 samples recovered after immersing in different polar solvents.

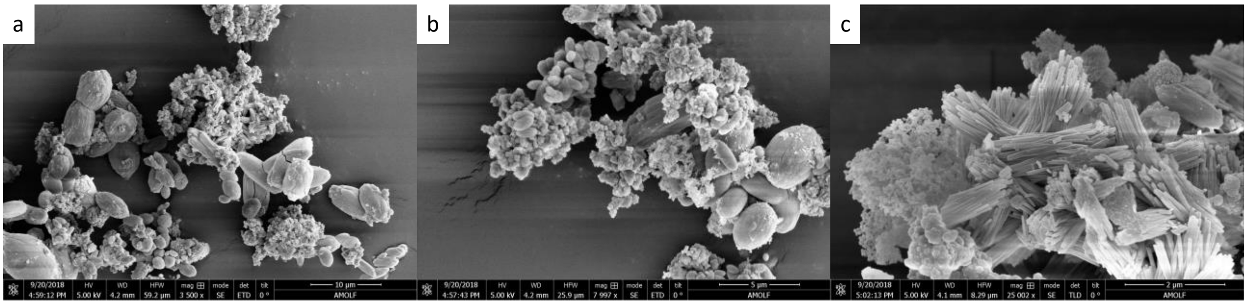

Fig. S4 SEM images of HIMS-74 particles immersed in methanol for 6 days with 3 times replenish of solvent.

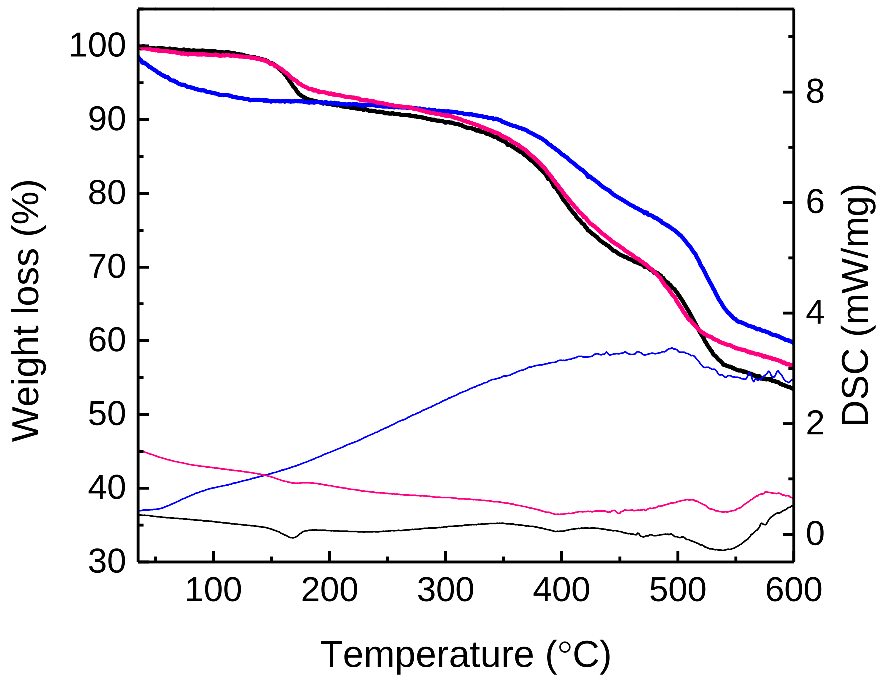

Fig. S5 TGA-DSC curves of as synthesized HIMS-74 (black) and the HIMS-74 samples recovered after exposure to methanol (blue) or acetone (pink).

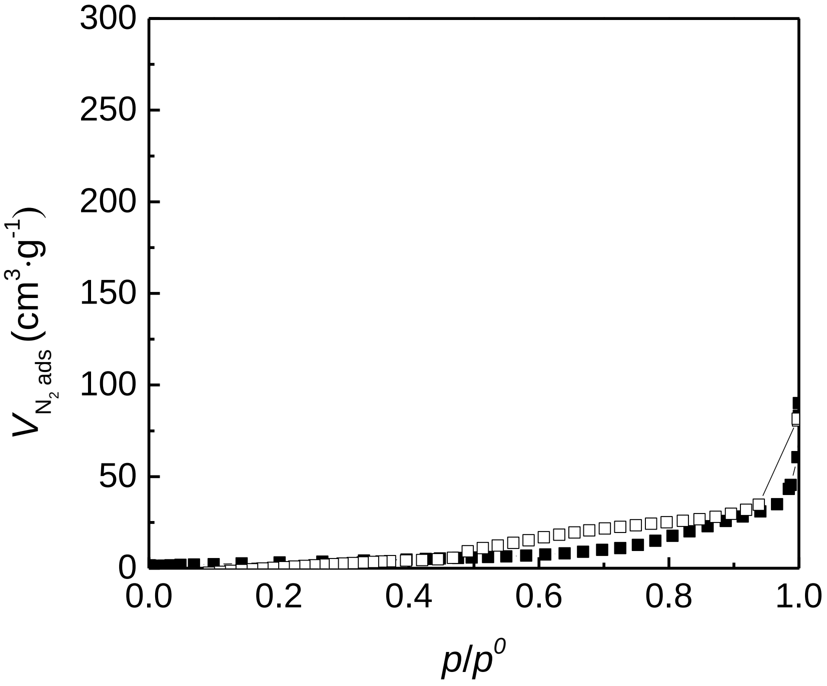

Fig. S6 N_2 adsorption isotherm for pretreated HIMS-74 recovered after immersion in MeOH at 77K.

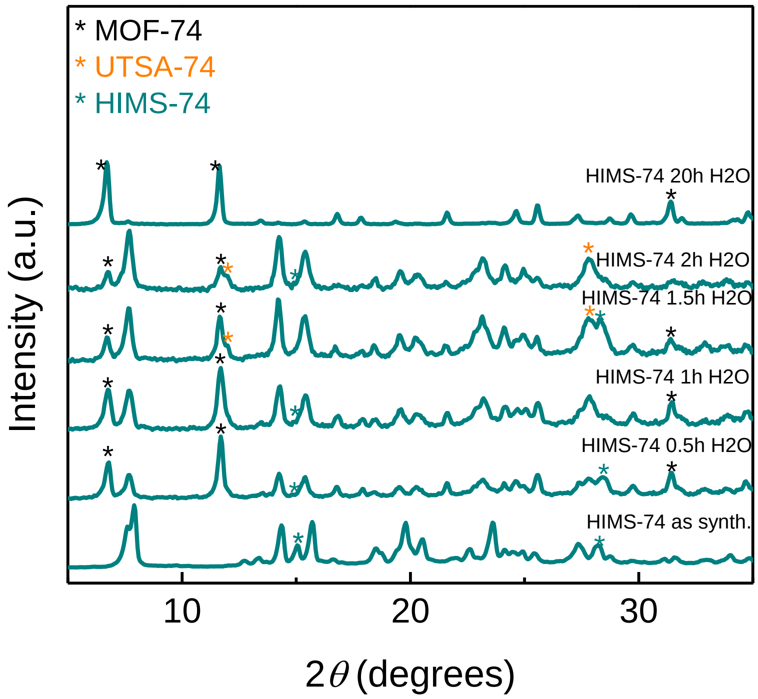

Fig. S7 PXRD patterns of the as synthesized HIMS-74 before and after immersion in H_2O at different stages showing its conversion to MOF-74. PXRD also indicates the presence of UTSA-74 phase.

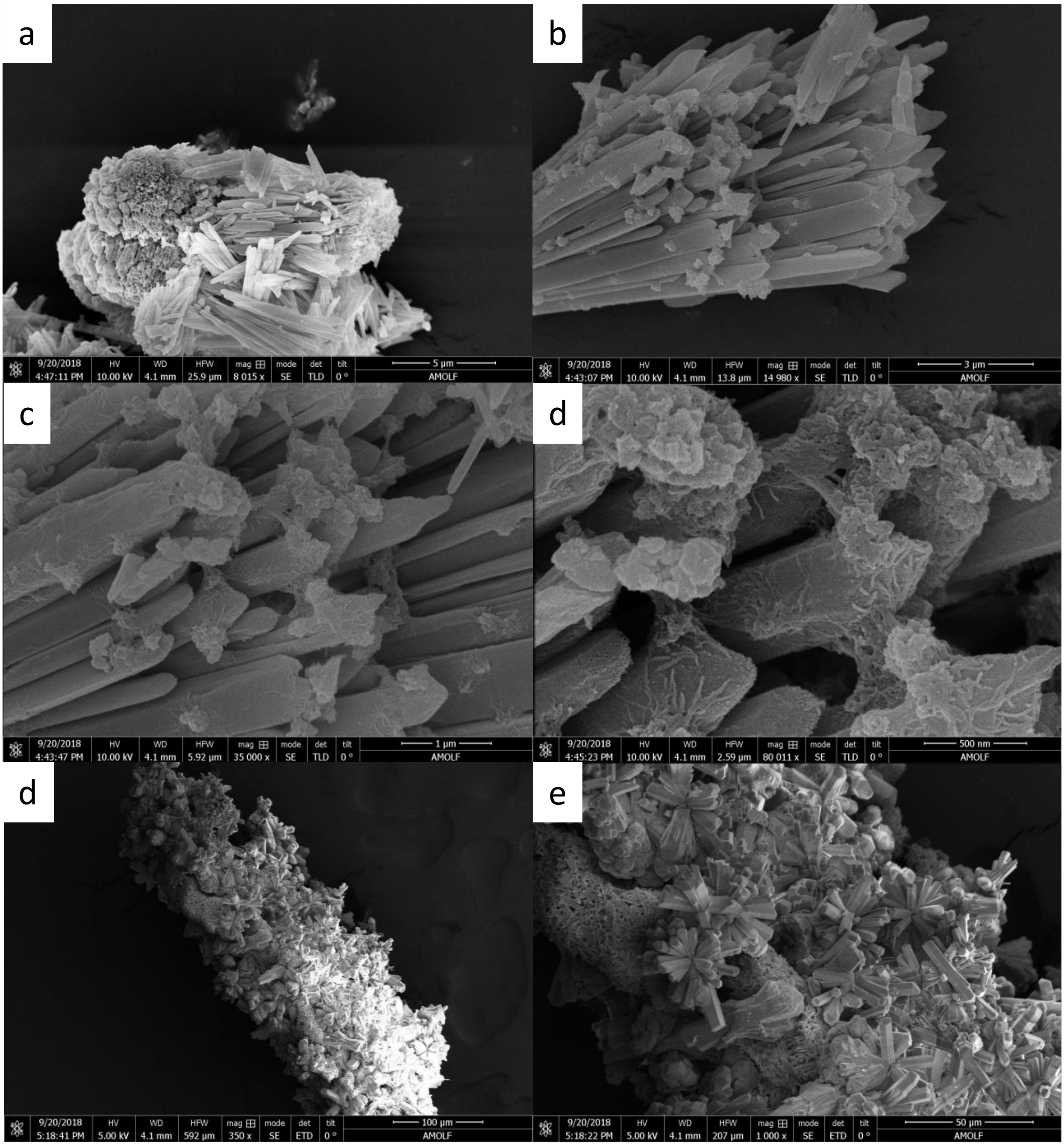

Fig. S8 Solvent-mediated isomerization of HIMS-74 to MOF-74 by immersion in H_2O and imaging of the crystals after 1.5h (a-d) and 20h (f-h) exposure time.

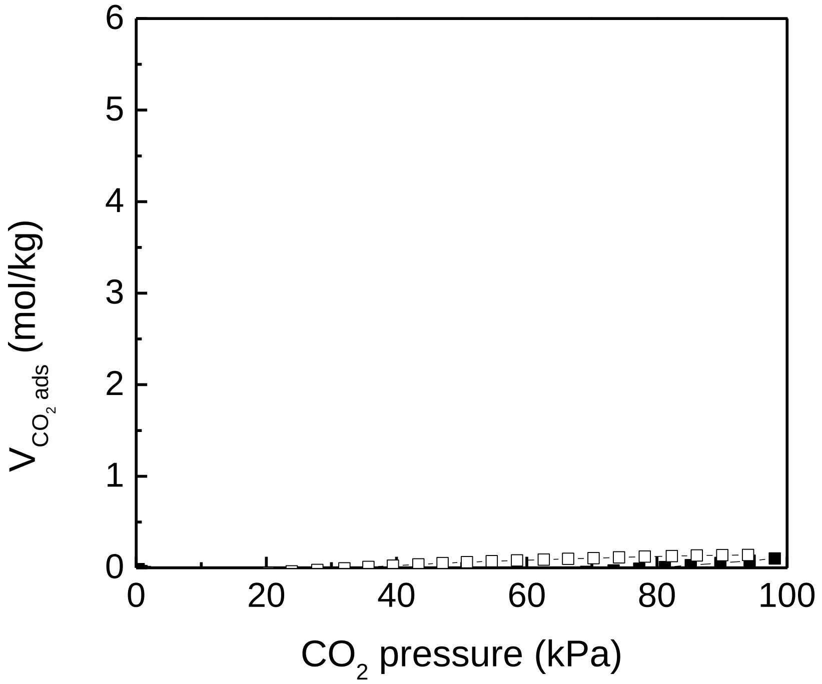

Fig. S9 CO_2 adsorption isotherm for activated HIMS-74 at 273K.

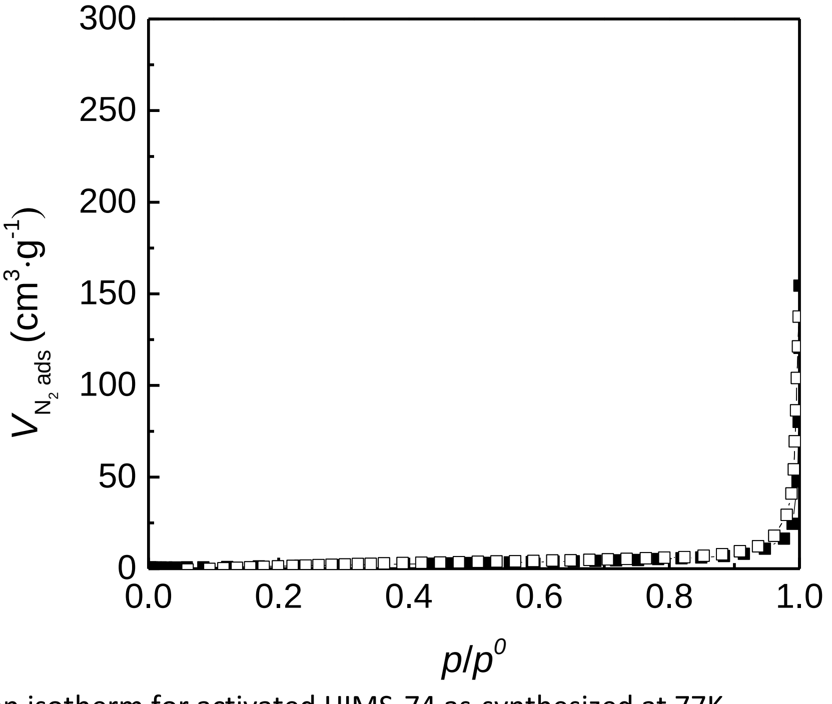

Fig. S10 N_2 adsorption isotherm for activated HIMS-74 as-synthesized at 77K.

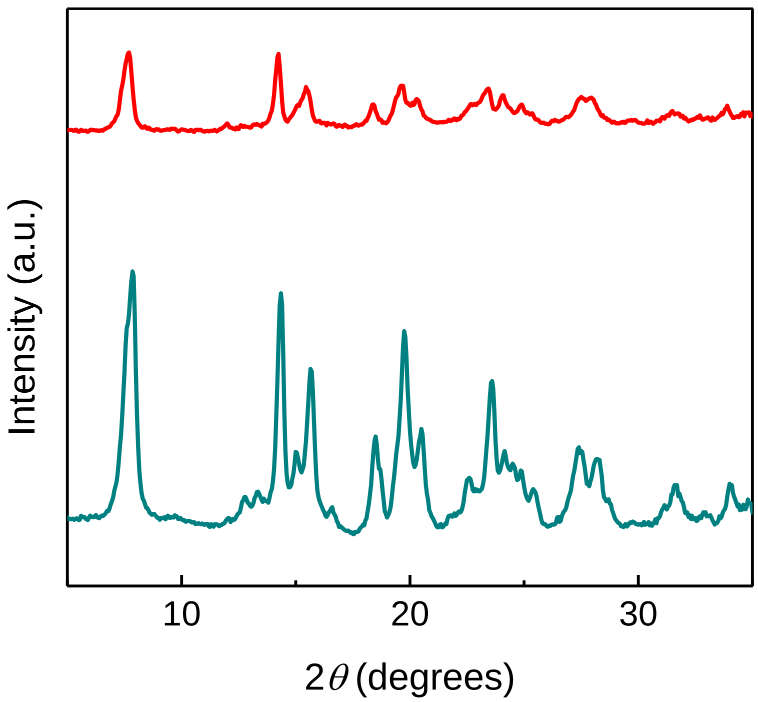

Fig. S11 PXRD patterns of the as synthesized HIMS-74 (cyan) and activated by vacuum heating with 1 °/min from room temperature to 150 °C and a hold of 2h.

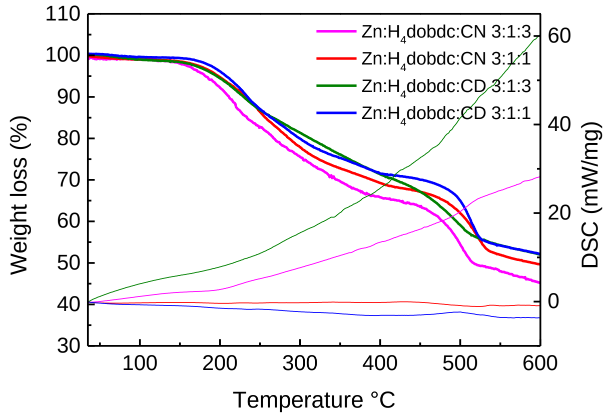

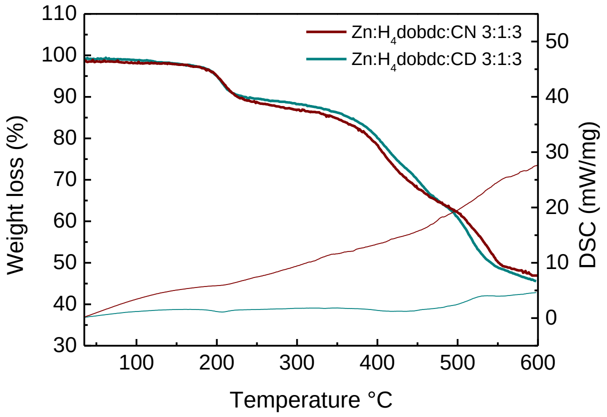

Fig. S12 TGA patterns of materials obtained in DMF (left) and materials obtained in NMP (right).

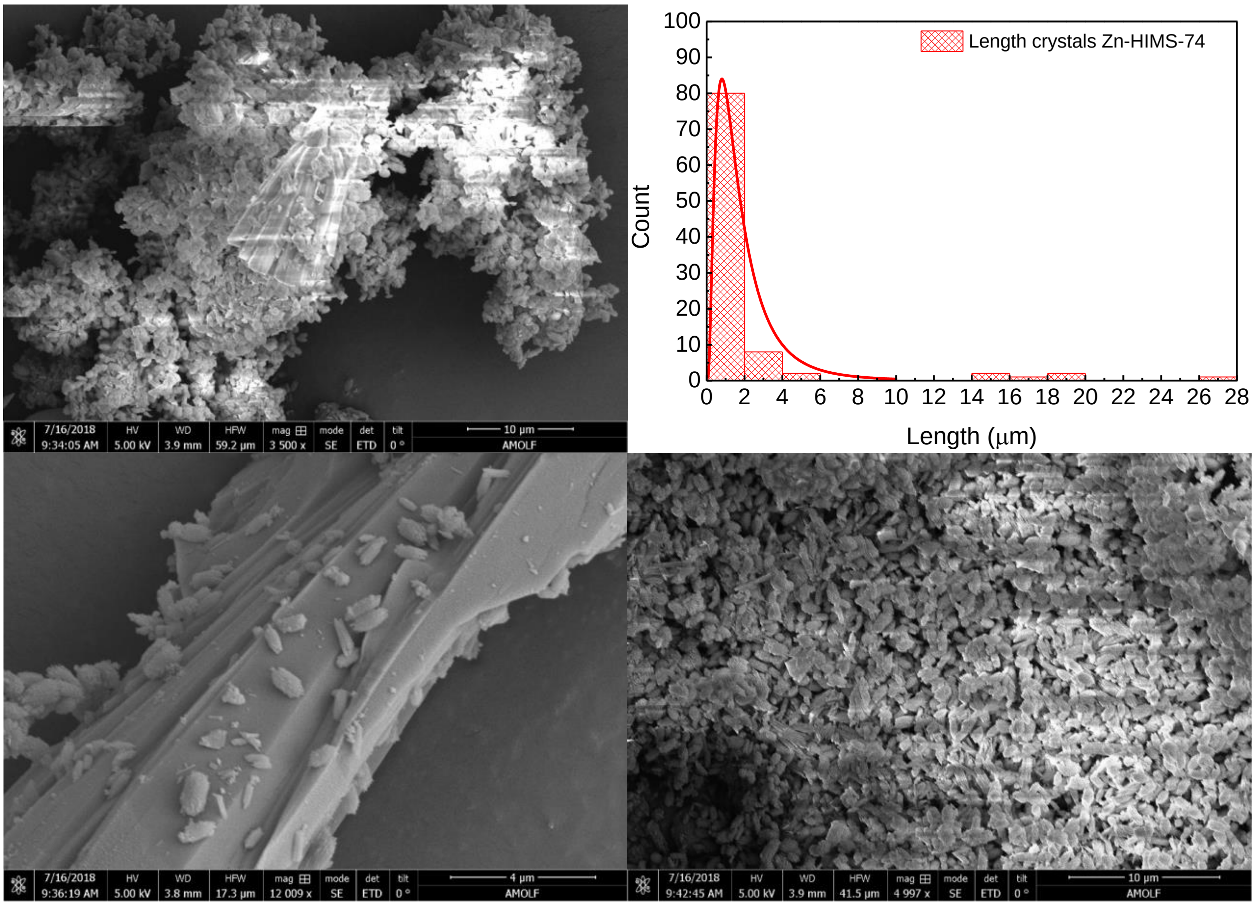

Fig. S13 SEM images of as synthesized HIMS-74 crystals and the corresponding histogram.

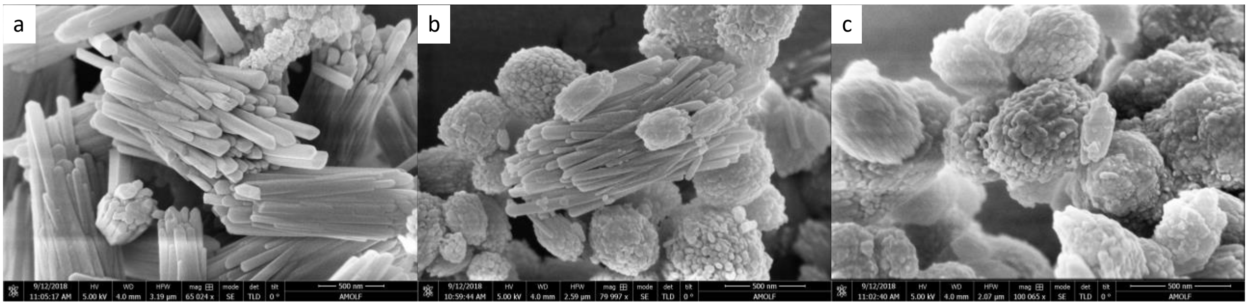

Fig. S14 SEM images of HIMS-74 particles synthesized at small scale (a) and large scale (b & c).

Table S1. Crystal Data and refinement information for complexes.

Complexes	**HIMS-74**	**UTSA-74***
Formula	$C_{44}H_{42}N_4O_{22}Zn_6$	$C_8H_2O_7Zn_2$
Formula weight	1371.16	340.88
Crystal system	Monoclinic	Trigonal
Space group	*P*2*1*/a	R-3c
a (Å)	14.030 (4)	22.970 (5)
b (Å)	23.660 (4)	22.970 (5)
c (Å)	15.820 (4)	15.910 (5)
α (°)	90	90
β (°)	113.39 (5)	90
γ (°)	90	120
Volume ($Å^3$)	4820(3)	7270(4)
Z	4	18
Dx (g/cm^3)	1.890	1.401
μ (mm^{-1})	3.212	3.164
Reflections Collected	7914	1943
Reflections Unique	7018	1817
R_1[a] [$I > 2\sigma(I)$]	0.0362	0.0560
wR_2[b]	0.0982	0.1746

[a]$R_1 = \Sigma||Fo|-|Fc|| / \Sigma|Fo|$,
[b]$wR_2 = \{\Sigma[w(|Fo|^2 - |Fc|^2)^2] / \Sigma[w(|Fo|^4)]\}^{1/2}$

*The formula and derived parameters are calculated without solvent.

References

1 D. Dubbeldam, S. Calero and T. J. H. Vlugt, *Mol. Simul.*, 2018, **44**, 653–676.
2 N. L. Rosi, J. Kim, M. Eddaoudi, B. Chen, M. O'Keeffe and O. M. Yaghi, *J. Am. Chem. Soc.*, 2005, **127**, 1504–1518.
